# Metabolic Dysfunction and Metabolic Management in Episodic Migraine

**DOI:** 10.1007/s11916-026-01500-9

**Published:** 2026-05-02

**Authors:** Delora E. Denney, Michael J. Marmura

**Affiliations:** 1 University of Colorado, Aurora, CO USA; 2 Department of Neurology, Jefferson Headache Center, Thomas Jefferson University, Philadelphia, PA 19107 USA

**Keywords:** Metabolic syndrome, Migraine, Obesity, Chronic pain, Exercise, Weight loss

## Abstract

**Purpose of Review:**

Evidence suggests a significant link between migraine and metabolic syndrome,, especially in those with chronic or refractory migraine. Weight loss and exercise may effectively prevent migraine. Here, we review the evidence behind exercise and weight loss as migraine treatments.

**Recent Findings:**

While metabolic syndrome and pain have long had a known association, only recently have strong studies demonstrated a higher risk of developing metabolic syndrome in people with migraine. Additionally, other sequelae of metabolic syndrome, obesity, hypertension, hypercoagulability, and diabetes also interplay with migraine. New medications like GLP-1 agonists may be a potential treatment for migraine as well as obesity.

**Summary:**

For patients with migraine, metabolic syndrome, and obesity, care should be taken when selecting prophylactic medications, favoring those without a propensity to induce weight gain. Additionally, there is strong evidence for weight loss and exercise for improving migraine outcomes.

## Introduction

Migraine is a complex neurological disorder influenced by genetic, medical, and environmental factors. Metabolic syndrome is a disorder that increases risks of cardiovascular disease and type 2 diabetes, as defined by hypertension, abdominal obesity, insulin resistance, and dyslipidemia. Metabolic syndrome and migraine may be linked, particularly as a risk factor for the evolution from episodic to chronic migraine. This review focuses on the available data demonstrating the influence of metabolic syndrome and obesity in those with chronic pain and migraine based on pathophysiological and clinical studies. It will also discuss treatment implications, including medication management, dietary recommendations, and exercise for migraine prevention.

## Metabolic Syndrome, Obesity, and Chronic Pain Disorders

Systemic disease, which can affect the entire body or multiple parts of the body, may cause headache. The International Classification of Headache Disorders-3 (ICHD-3) contains an appendix listing for headache attributed to a systemic disorder. Several disorders of homeostasis known to cause headache include several hypertensive disorders, thyroid disease and sleep apnea [1]. Metabolic syndrome is an example of a systemic disease with the potential to worsen headache, due to chronic inflammation, which is important in the development of chronic pain [2]. The manifestation or worsening of headache in the context of a systemic disease requires thorough evaluation to determine if headache is due to a secondary etiology [3].

There are two different sets of criteria commonly used to diagnose metabolic syndrome: (1) the National Cholesterol Education Program Adult Treatment Panel III (NCEP ATP III) criteria and (2) the International Diabetes Federation (IDF) criteria. The NCEP ATP III requires 3 of the 5: waist circumference > 40 inches in men and > 35 inches in women, fasting glucose ≥ 100 mg/dl or hyperglycemia treatment, triglycerides ≥ 150 mg/dl or hypertriglyceridemia treatment, HDL < 40 mg/dl in men and < 50 mg/dl in women, blood pressure > 130 mmHg systolic or > 85 mmHg diastolic or hypertension treatment. The IDF requires waist circumference of ≥ 94 cm in men and ≥ 80 cm in women plus 2 of the 4: fasting glucose ≥ 100 mg/dl, triglycerides ≥ 150 mg/dl or hypertriglyceridemia treatment, HDL < 40 mg/dl in men and < 50 mg/dl in women, blood pressure > 130 mmHg systolic or > 85 mmHg diastolic or hypertension treatment [4].

Metabolic syndrome causes a state of inflammation and increased thrombotic risk, resulting in an increase of markers such as C-reactive protein (CRP), tumor necrosis factor-alpha (TNF-α), fibrinogen, and interleukin-6 (IL-6) as well as plasminogen activator inhibitor-1 (PAI-1), which is prothrombotic [5]. One proposed contributory mechanism for this inflammation is that adiponectins, such as leptin, decrease as body mass index (BMI) increases, resulting in a decrease in adiponectin’s anti-inflammatory effects [6].

Metabolic syndrome is strongly linked with chronic pain [7], especially neuropathic pain such as fibromyalgia [8]. Two factors that may influence pain perception in those with metabolic syndrome are endocannabinoids and the gut microbiome. Metabolic syndrome may disrupts endocannabinoid signaling. interfering with pain and weight regulation [9]. Metabolic syndrome alters the composition of the gut microbiome and changes pain perception in syndromes like fibromyalgia and osteoarthritis. Patients who meet some criteria for metabolic syndrome have microbiomes poor in fiber, which is also a feature of microbiomes found in patients with fibromyalgia and osteoarthritis [10].

In a study of women with fibromyalgia compared to healthy controls, those with fibromyalgia were 5.56 times more likely to meet NCEP ATP III criteria for metabolic syndrome. Women with fibromyalgia were also found to have increased triglycerides, total cholesterol, hemoglobin A_1c_ levels, blood pressure, and waist measurements that remained significant even after controlling for age and BMI as compared to the healthy controls [7]. This study also had strict exclusion criteria; those with any other type of chronic pain or other disorder that could increase risk for metabolic syndrome such as diabetes mellitus (DM) were excluded. A study of rheumatological diseases, such as rheumatoid arthritis, showed that patient-rated pain and disease activity measures increased with the number of criteria met for metabolic syndrome [11]. A study looking at metabolic syndrome and low back pain found that patients with both had a statistically significantly increased low back pain disease duration [12].

The prevalence of chronic pain increases in a linear fashion among obese individuals [13], particularly in the morbidly obese. In the elderly, chronic pain is more common in the severely obese, even when adjusting for factors such as age, sex, education, mood disorders, and other medical disorders [14].

## The Prevalence of Metabolic Syndrome in Migraine

Metabolic syndrome is marked by central obesity, hyperglycemia, hyperlipidemia, and hypertension which are risk factors for developing diabetes mellitus (DM) and cardiovascular disease (CVD). Metabolic syndrome has also been linked to several nervous system disorders such as Parkinson’s disease and dementia [15, 16].

Andreeva et al. conducted a systematic review of studies published between 2009 and 2017 evaluating associations between migraine and metabolic syndrome. A handful of studies have investigated the presence of migraine in those with metabolic syndrome, showing that a large range of 2.8–58.3% had migraine. In studies of those with migraine, 12–33% have metabolic syndrome, but the variability of study designs and analysis make it difficult to generalize these findings [17]. The best evidence comes from a cohort study in Norway showing a statistically significant increased risk of metabolic syndrome in those with migraine, with an even higher risk in those with aura [18]. A newer study of 240 migraine patients in Iran showed an overall prevalence of metabolic syndrome at 16.25%, with higher prevalence in those with more severe disease [19].

A 2022 case-control study of patients with migraine vs. healthy controls using statistical power calculations and satisfying the requirements for such also showed a significant link between migraine and metabolic syndrome at 46.7% in those with migraine compared to16.7% in for age and sex matched controls. Those with migraine also had a statistically significant increase in waist circumference, insulin resistance, and serum insulin levels. No differences were found in triglyceride levels, fasting blood glucose levels, or high-density lipoprotein (HDL). Insulin resistance and increased adipose tissue both contribute to a pro-inflammatory state that can lead to the development of or exacerbate migraine and/or metabolic syndrome [20].

## Metabolic Syndrome Sequalae: Diabetes Mellitus, Hypertension, and Hypercoagulability

Metabolic syndrome confers a fivefold increased risk of developing DM [21]. The link between DM and migraine is less clear. A systematic literature review of studies on migraine and diabetes from 2012 to 2019 was conducted by Hosseinpour et al. [22]. The hazard rate in one study for developing migraine was 1.06 overall, but the rate was higher for those without aura with a rate of 1.13 [23]. One study showed that migraine and Type 1 DM were inversely related, implying DM treatment might also offer a protective effect against migraine [24]. A more recent meta-analysis suggested that diabetes does not increase the risk of migraine, but that a history of migraine may increase diabetes risk [25].

Hypertension is part of the criteria for metabolic syndrome, conferring an increased risk for CVD. Headache is a common symptom of hypertension, and hypertension is included in the ICHD-3 as “headache attributed to arterial hypertension [1].” While hypertension can trigger headache, there may be a correlation between migraine and essential hypertension [26]. Elevated diastolic blood pressure is associated with migraine in the majority of studies to date, forming a bidirectional relationship where each confers increased risk of the other [27].

Hypercoagulability has been linked with metabolic syndrome [28] and migraine, specifically migraine with aura. Several mechanisms have been proposed for why those with migraine have an increased risk for thrombosis. The first is a higher level of estrogen, whether natural or induced with oral contraception; as estrogen increases, so does clotting risk. The second is the activation of platelets. Due to increasing cycloxagenase-2 (COX-2), an inflammatory molecule, migraine has a known association with decreased levels of endocannabinoids, which normally functions to prevent platelet activation and encourage vasodilation, therefore platelets are activated pathologically in those with migraine. Those with migraine also have higher levels of von Willebrand factor expressed from endothelial cells, which can promote clotting and increases stroke risk [29].

## Obesity as a Risk Factor for Idiopathic Intracranial Hypertension and Migraine

Idiopathic intracranial hypertension (IIH), formerly known as pseudotumor cerebri, is a distinct headache diagnosis with a pathophysiology largely attributable to obesity. It classically presents in young, obese females with papilledema, possible vision loss, and headache, usually with migraine features [30]. Weight loss is a mainstay of treatment and is practically curative in most cases, giving strong support to IIH’s relationship to obesity [31]. However, it should be noted that in some cases of IIH, the headache remains after effective treatment of IIH and responds well to migraine therapy [32].

People with obesity, particularly women younger than 55, have a 21% increased risk of migraine. For those with episodic migraine, obesity is a risk factor for progression to chronic migraine. Obesity is also associated with more disability due to migraine and increased attack frequency as BMI increases [33] and with migraine disease duration [34]. Concomitantly, losing weight is associated with decreased migraine attack frequency as well as decreased headache severity and overall disability in adolescents [35]. Elevated levels of calcitonin gene-related peptide (CGRP), a peptide known to be involved in migraine pathogenesis [36], have also been observed in obese women. Additionally, the hormone amylin, which promotes satiety, is elevated in obese individuals and has been demonstrated to incite migraine attacks. possibly due to its similarity with the CGRP molecule [37].

It is also worth noting that migraine can be a risk factor for obesity due to the very nature of being in pain, which can perpetuate inactivity and lack of exercise. Additionally, migraine is well known to be comorbid with psychological disorders like depression with a linked with further risk of weight gain. Therefore, it is reasonable to propose that the relationship between migraine and obesity can be bidirectional [38].

## Treatment Considerations

Given the high prevalence of metabolic syndrome and obesity in migraine, clinicians should encourage healthy lifestyles and behavior as part of their overall management. Behavioral protective factors for migraine include adequate sleep, stress management, healthy diet, and exercise [39]. These interventions may treat or prevent the development of chronic migraine [40]. For specialists, this also means recommending appropriate primary care screening, referrals to specialists for problems such as sleep disorders or weight management and obtaining vital signs for in-person visits. This may include discussion of how inadequately-treated migraine increases risks of obesity and poor health, possibly due to physical inactivity, medications, or other medical factors.

## Pharmacological Management

Weight gain is a common side effect of medications used for migraine prevention, including antidepressants, beta-blockers, and anticonvulsants [41]. Weight gain, or fear of weight gain, is among the most common reasons to discontinue or reject preventive treatment in clinical practice [42], even when the treatment is effective [43]. When choosing migraine prevention, it is important to honestly review the potential risks and benefits of treatment and consider strategies to mitigate weight gain.

Clinicians frequently use antidepressants off-label for headache prophylaxis. In an observational study, amitriptyline, a tricyclic antidepressant frequently utilized for headache prophylaxis, was more likely to cause weight gain at a dose of 20–40 mg per day compared to propranolol 80–160 mg, and valproate 600 mg, with about 60% of patients gaining weight over 6 months of treatment [44]. Rates of weight gain are also significant in other studies using similar medications such as nortriptyline [45]. Venlafaxine and duloxetine are serotonin–norepinephrine reuptake inhibitors (SNRIs) with some evidence for effectiveness in migraine prophylaxis [46]. While less likely to cause immediate weight gain compared to amitriptyline, SNRIs are associated with the potential for weight gain from long-term use [47].

Several other drugs used for migraine prevention also increase the risk of weight gain. Beta-blockers such as propranolol, atenolol, or metoprolol are associated with weight gain in a minority of subjects in cardiac clinical studies, and among those using them for migraine prophylaxis [48]. Patients using anticonvulsants such as divalproex sodium and gabapentin are also more likely to have weight gain [49]. The use of the anticonvulsant topiramate is more likely to cause weight loss compared to placebo and other treatments, but the likelihood of weight loss is not associated with factors such as high body mass [50].

The impact of CGRP and drugs that target CGRP on weight gain and metabolic syndrome is unclear. One early study demonstrated increased CGRP levels in obese women, which did not improve after weight loss [51], but a more recent study found that successful weight loss surgery in migraine patients improved migraine frequency and lowered serum CGRP levels [52]. In preclinical models, blocking CGRP does not affect lipid metabolism or glucose [53], and most anti-CGRP medications did not affect weight compared to placebo in clinical trials. However, a safety analysis reported dose- and treatment-duration-dependent weight loss in a minority of subjects using atogepant, an oral anti-CGRP preventive medication, in their pivotal trials [54].

## Treatment of Obesity in Migraine

A meta-analysis by Di Vencenzo et al. in 2020 showed that weight loss improves outcomes in migraine [55]. Specifically, weight loss in general leads to decreased pain intensity, attack duration, and frequency. Impressively, the authors found no relation of improvement to weight at baseline, how much weight was lost, nor the method by which the weight was lost (surgical vs. non-surgical) [55].

Glucagon-like peptide-1 (GLP-1) agonists, approved for the treatment of obesity and diabetes, may benefit those with chronic pain [56]. In one study a GLP-1R agonist, liraglutide activated GLP-1R in the trigeminal nucleus caudalis and decreased the expression of CGRP [36]. GLP-1 activates interleukin-10 (IL-10), which downregulates inflammatory mediators like TNF-a which are implicated in migraine [36]. Another study found that GLP-1 levels are lower in migraine compared with controls [57].

A small randomized controlled trial in patients with IIH and a BMI ≥ 30 kg/m2 showed that GLP-1 agonists conferred a statistically significant reduction in monthly migraine days, with around 75% of patients having a 50% reduction in monthly migraine days 6 months after beginning treatment. However, the treatment group also had statistically significant weight loss which could be a confounding variable in headache reduction. The main limitation of this study is that the treatment group only included 13 patients [31]. Another report describes the emergence of IIH in a patient after abruptly stopping GLP-1 treatment [58].

## Exercise for Migraine Prevention

Regular exercise can prevent migraine either on its own or in combination with medication [59]. A meta-analysis and systematic review by Lemmens et al. including 6 studies on exercise and migraine concluded that aerobic exercise three days a week leads to a mean reduction of 0.6 ± 0.3 migraine days/month as well as decreased migraine attack duration and intensity [60]. In another study, exercise for 40 min three days per week, showed similar efficacy as topiramate in migraine prevention [61]. In patients with chronic migraine, combining an exercise regimen with amitriptyline prophylaxis led to a significant reduction over treatment alone in headache intensity, duration, and frequency [62]. More intensive exercise bestows a greater reduction in migraine days. However, in patients who experience exercise-induced migraine, lower-intensity exercise such as yoga or tai-chi also decreases headache frequency [63, 64]. The proposed mechanism of action is modification of inflammatory mediators like CRP, cytokines (CGRP, substance P) and adipocytokines (TNF-a, IL-6) which have also been implicated in migraine [65] Table 1.

**Table 1:** Metabolic syndrome diagnostic criteria comparison^4^

CEP ATP III	CEP ATP III	IDF	IDF
Counts towards 3 of 5 required	waist circumference > 40 inches in men and > 35 inches in women	Required	waist circumference of ≥ 94 cm in men and ≥ 80 cm in women
Counts towards 3 of 5 required	fasting glucose ≥ 100 mg/dl or hyperglycemia treatment	Counts towards 2 of 4 additionally required	fasting glucose ≥ 100 mg/dl
Counts towards 3 of 5 required	triglycerides ≥ 150 mg/dl or hypertriglyceridemia treatment	Counts towards 2 of 4 additionally required	triglycerides ≥ 150 mg/dl or hypertriglyceridemia treatment
Counts towards 3 of 5 required	HDL < 40 mg/dl in men and < 50 mg/dl in women	Counts towards 2 of 4 additionally required	HDL < 40 mg/dl in men and < 50 mg/dl in women
Counts towards 3 of 5 required	blood pressure > 130 mmHg systolic or > 85 mmHg diastolic or hypertension treatment	Counts towards 2 of 4 additionally required	blood pressure > 130 mmHg systolic or > 85 mmHg diastolic or hypertension treatment

## Conclusions

Systemic diseases, such as metabolic syndrome, can co-occur with other pain and inflammatory disorders like migraine. The relationship is likely bi-directional, as demonstrated by women with fibromyalgia having an increased risk of developing metabolic syndrome even after controlling for factors like BMI. Obesity negatively impacts pain syndromes like migraine and contributes to the development of metabolic syndrome Table 2.

**Table 2:** Migraine prophylactic treatments and their relation to weight

Can cause weight gain	Weight neutral	Can cause weight loss
Tricyclic antidepressants	Calcitonin gene related peptide antagonists	Topiramate^42^
Serotonin–norepinephrine reuptake inhibitors^45^	Lisinopril, Candesartan	Atogepant^52^
Beta-blockers		Exercise
Anticonvulsants^46^		

A variety of studies have linked metabolic syndrome and migraine bidirectionally, although earlier studies were not as robust as more recent literature, which has focused on the prevalence of metabolic syndrome in those with migraine. The sequalae of metabolic syndrome, DM, hypertension, and hypercoagulability, also have interesting relationships with migraine. The most significant association between migraine and hypertension is elevated diastolic blood pressure. Hypercoagulability, mainly linked to migraine with aura, can also result from metabolic syndrome, with a mutually proposed mechanism of increased estrogen.

Migraine and obesity also exist in a bidirectional relationship. Obesity has been shown to increase migraine disease burden, whereas weight loss, even if modest, can positively impact migraine. However, migraine itself can contribute to the development of metabolic syndrome due to inactivity, depression and the use of medications which cause weight gain. The relationship between migraine and mental health, as manifested by mood disorders including anxiety and depression, has been well documented [66]. However, it is worth considering the side effect profile of common first-line migraine preventives and their possible contribution to weight gain. Newer anti-CGRP medications may be the best option for patients already struggling with obesity or noted to be gaining weight on their current migraine prophylactic therapy. GLP-1 agonists may also be a good option in the future if further evidence supports their safety and effectiveness. Additionally, weight loss can be an excellent prophylactic treatment in its own right and should be considered an integral part in any migraine treatment regimen, especially for overweight or obese patients.

Providers who treat migraine should consider the toll of the disorder on the physical well-being of patients. This may include recommending exercise for patients with migraine, choosing medications less associated with weight gain, and evaluating for markers of metabolic syndrome, such as increased blood pressure, dyslipidemia, and insulin resistance.

## Key References

- Ali M, Hussein M, Magdy R, et al. The potential impact of insulin resistance and metabolic syndrome on migraine headache characteristics. BMC Neurol. 2022;22(1):422.
  - ○ People with migraine are statically significantly more likely to have insulin resistance. Those with migraine and insulin resistance have more severe migraine attacks than those without insulin resistance.
- Jahromi SR, Martami F, Morad Soltani K, Togha M. Migraine and obesity: what is the real direction of their association? Expert review of neurotherapeutics. 2023;23(1):75–84.
  - ○ Explores the evidence for the link between migraine and obesity and confounding factors that require more investigation.
- Etefagh HH, Shahmiri SS, Melali H, et al. Bariatric surgery in migraine patients: CGRP level and weight loss. 2022;32(11):3635–40.
  - ○ Weight loss can not only decrease migraine attack frequency and severity but also degreases CGRP levels.

## Abbreviations

ICHD: International Classification of Headache Disorders
NCEP ATP III: National Cholesterol Education Program Adult Treatment Panel III
IDF: International Diabetes Federation
CRP: c-reactive protein
IL-6: interleukin-6
TNF-α: tumor necrosis factor-alpha
BMI: body mass index
DM: diabetes mellitus
CVD: cardiovascular disease
IIH: Idiopathic Intercranial Hypertension
CGRP: calcitonin gene related peptide
GLP-1: Glucagon-like peptide-1
GLP-IR: Glucagon-like peptide-1 receptor
